# Bulk metal concentrations versus total suspended solids in rivers: Time-invariant & catchment-specific relationships

**DOI:** 10.1371/journal.pone.0191314

**Published:** 2018-01-17

**Authors:** Touraj Nasrabadi, Hermann Ruegner, Marc Schwientek, Jeremy Bennett, Shahin Fazel Valipour, Peter Grathwohl

**Affiliations:** 1 Graduate Faculty of Environment, University of Tehran, Tehran, Iran; 2 Center for Applied Geosciences, University of Tübingen, Tübingen, Germany; Natural Environment Research Council, UNITED KINGDOM

## Abstract

Suspended particles in rivers can act as carriers of potentially bioavailable metal species and are thus an emerging area of interest in river system monitoring. The delineation of bulk metals concentrations in river water into dissolved and particulate components is also important for risk assessment. Linear relationships between bulk metal concentrations in water (*C*_*W*,tot_) and total suspended solids (TSS) in water can be used to easily evaluate dissolved *(C*_*W*_, intercept) and particle-bound metal fluxes *(C*_*SUS*_, slope) in streams *(C*_*W*,*tot*_ = *C*_*W*_ + *C*_*SUS*_ TSS). In this study, we apply this principle to catchments in Iran (Haraz) and Germany (Ammer, Goldersbach, and Steinlach) that show differences in geology, geochemistry, land use and hydrological characteristics. For each catchment, particle-bound and dissolved concentrations for a suite of metals in water were calculated based on linear regressions of total suspended solids and total metal concentrations. Results were replicable across sampling campaigns in different years and seasons (between 2013 and 2016) and could be reproduced in a laboratory sedimentation experiment. *C*_*SUS*_ values generally showed little variability in different catchments and agree well with soil background values for some metals (e.g. lead and nickel) while other metals (e.g. copper) indicate anthropogenic influences. *C*_*W*_ was elevated in the Haraz (Iran) catchment, indicating higher bioavailability and potential human and ecological health concerns (where higher values of *C*_*SUS*_/*C*_*W*_ are considered as a risk indicator).

## Introduction

River bed sediment pollution is a long-standing area of environmental concern [1,2]. Toxic metals/metalloids are readily transported by carriers (e.g. suspended solids) towards lakes, estuaries, or oceans. In contrast to many other frequently studied pollutants (e.g. organic compounds) they are non-degradable [3,4]. Although environmental quality standards for many metals (e.g., nickel, lead) are based on dissolved or bioavailable concentrations [5], suspended sediments may contribute to mass flux and relocation of metals. Therefore, suspended particles as vectors of potentially bioavailable metal species have been extensively studied in recent years [6–9]. Monitoring of suspended and dissolved metal fluxes is time-consuming, expensive and labor-intensive, as it generally includes multi-stage multi-sample processing schemes and speciation analysis [10]. Easy-to-measure proxies like Total Suspended Solids (TSS) and turbidity have been used recently for concentration and/or flux estimations of a wide range of pollutants, including persistent organic pollutants [11,12], pesticides [13], and phosphorus [14]. Schwientek et al. [11] and Ruegner et al. [15] reported very robust correlations between total concentrations of polycyclic aromatic hydrocarbons (PAH) with TSS or turbidity in tributaries of the River Neckar in southwest Germany. Such correlations were observed for polycyclic aromatic hydrocarbons in several catchments in Germany [11,16] and found to be constant over several years and seasons [17]. This allowed freely dissolved and particle-bound PAH concentrations and assessment of total PAH fluxes to be calculated.

In comparison with organic pollutants, metals have not been widely included in such proxy-aided estimations. Recently, Beltaos and Burrell [8] showed correlations between total concentrations of 17 metals and TSS in the Saint John River, Canada, during the ice melting period, when extremely high loads of suspended solids are transported. Nasrabadi et al. [18] reported robust linear correlations between TSS/turbidity and total concentrations of metals (Ni, Pb, Cd, Cu, Zn, Co, As, and Sr) in a single monitoring campaign in the Haraz River in the southern Caspian Sea Basin, an area affected by intensive sand and gravel mining activities. In their study, pollutant transport (dissolved plus particle-bound concentrations) could be easily monitored over time using regular TSS measurements once calibrated against *C*_*W*,*tot*_ for a given catchment. Continuous monitoring of pollutant fluxes using online turbidity sensors resulted in robust relationships between TSS and turbidity in the catchments of interest.

A broader investigation of these proxy (TSS/turbidity)-based methods across catchments differing in land-use, geology and climate has not yet been conducted. In addition, detailed investigations on the variability of metal concentrations on suspended solids over time in catchments have not been performed. The specific goals of this study were to i) establish relationships between total metal concentrations and total suspended sediments, ii) compare different catchments/land use/geology (Germany and Iran), and iii) check stability and reproducibility of metal-TSS relationships over time (e.g. different events/seasons/laboratory methods). This work follows earlier studies which showed time invariant but catchment specific behavior for polycyclic aromatic hydrocarbons [11,15].

## Materials and methods

### Suspended sediment sampling: Concept and theory

Suspended sediments represent a mixture of particles present in a river system coming from different sources in upstream areas [19]. These sediments are typically mobilized during high discharge events or anthropogenic disturbances such as dredging or mining. Rivers are integrators of catchment processes and suspended sediments are much less affected by local heterogeneity commonly associated with grab sediment samples. Furthermore, bulk water samples may be easily analyzed for total metal concentrations (water plus suspended sediment). The total concentration of metals and other particle-associated pollutants in bulk water samples consists of both the dissolved and particle-bound fraction:

$$C_{W,tot}=C_{W}+C_{SUS}\;TSS$$ (1)

*C*_*W*,*tot*_, *C*_*W*_, *C*_*SUS*_ and *TSS* denote the total and dissolved concentrations of the analyte in river water, its concentration on suspended particles and the suspended particle concentration in river water, respectively. Thus, in a plot of *C*_*W*,*tot*_ versus *TSS*, *C*_*SUS*_ corresponds to the slope of a linear regression while *C*_*W*_ corresponds to its intercept. These assumptions are valid if the dissolved and particle-bound concentrations of pollutants remain generally constant (e.g. during an event or depending on sampling location).

### Sites and sampling campaigns

In order to investigate the applicability of linear TSS-total metal concentrations relationships four study catchments were selected: The relatively small Ammer, Goldersbach, and Steinlach catchments in SW-Germany, which were already well investigated in terms of PAH concentrations in suspended sediments, and the larger Haraz catchment in Iran, where metal concentrations in river bed sediments have already been studied at several locations [18]. Ammer, Steinlach and Goldersbach catchments differ mainly in terms of land-use [20] The Haraz catchment, on the other hand, is distinct with regard to geology, climate, and land-use. None of sampling locations in the selected catchments were within a protected area or private land and no specific permission was required for river water sampling in the framework of this study. It is also confirmed that the field investigations did not interfere with endangered or protected species. Sampling campaigns were conducted, which included the measurement of total, dissolved and particle-bound metal concentrations; results were compared to earlier data and geochemical background concentrations.

**Fig 1:**
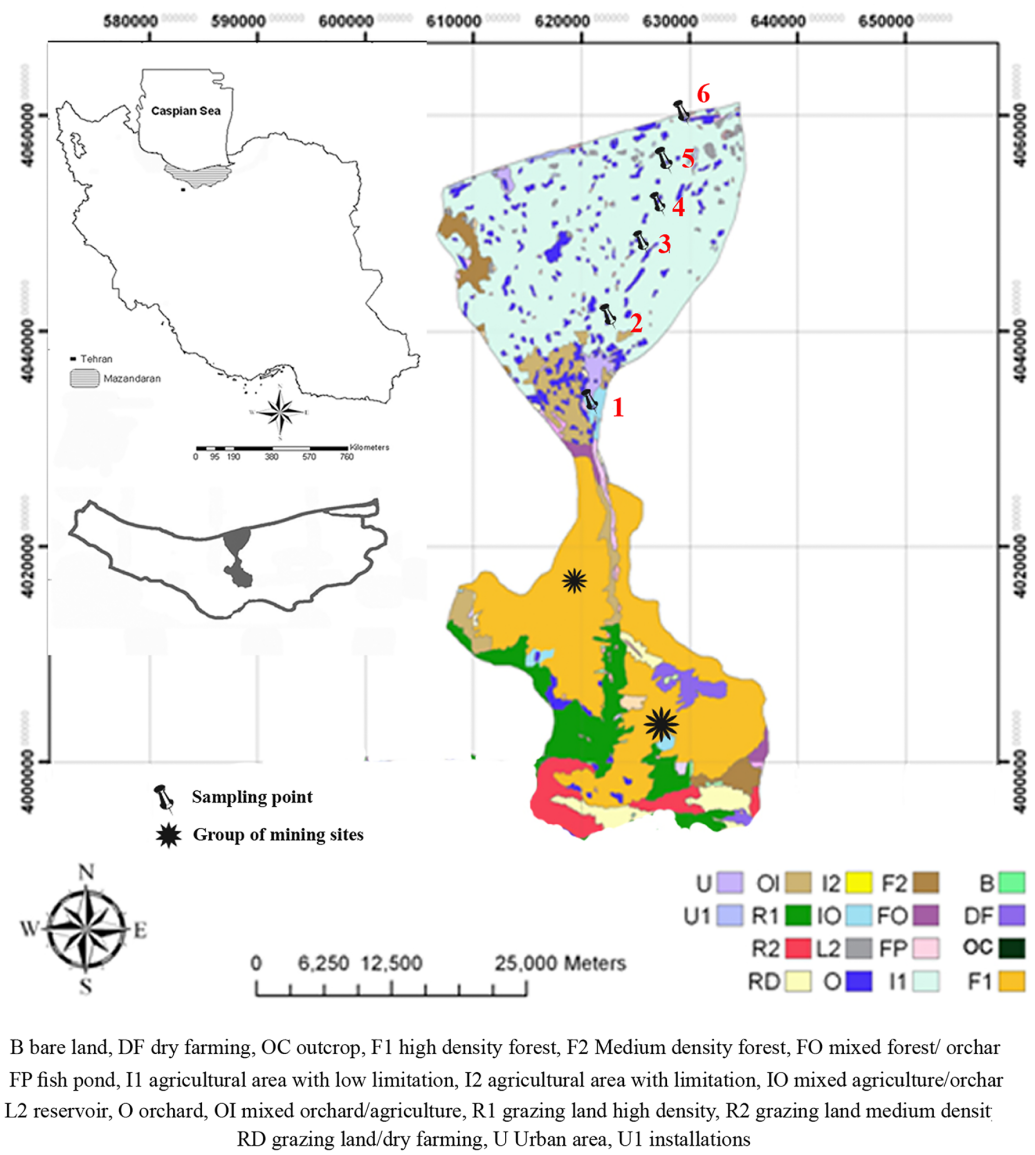
Map of the lower Haraz Basin with major land use as well as the sampling locations; numbers indicate sampling sites. Reprinted from [18] under a CC BY license, with permission from Elsevier, original copyright 2016.

The **Haraz River**, with a total drainage area of around 4,060 km^2^ and an approximate main channel length of 185 km, stretches from the Alborz mountain range about 3500 m above sea level (asl) in northern Iran towards the southern coastline of the Caspian Sea. The river slope changes from 13% in the mountainous headwaters to less than 0.1% in the lower areas which have been considered for analysis in this study (Fig 1). The mean annual flow is estimated to be 30 m^3^ s^-1^ [21]. However, recent local droughts have led to decreasing discharges. The geology of the catchment is described by Davidson et al. [22]. In brief, major formations are the sandstone-shale-type Shemshak Formation, the Lar and Delichi carbonates, and the Eocene Karadj formation that largely comprises submarine tuffs [23]. Hydrothermal springs related to former and ongoing volcanic activities are present in central parts of the basin. Coal-rich outcrops in the southern and central parts of the catchment have been exploited for decades. Sand and gravel mining activities to provide construction materials have profoundly affected downstream water quality. With more than forty active mining sites (coal, limestone, sand and gravel, etc.), the basin is considered to be heavily affected by anthropogenic activities which may have led to a release of dissolved and particle-bound metals either from anthropogenic or/and geogenic sources. Due to these ongoing upstream activities, water is often turbid even during low flow conditions. The catchment is mainly covered by grasslands, dense forests, rice paddies and urban settlements (Fig 1). The intensity of urban, rural, industrial and agricultural land use increases downstream and, consequently, the load of discharged wastewaters and run-off also increases.

To assess several potential metal pollution sources, six sampling locations were selected in the lower part of the Haraz catchment. The sampling campaign was performed in March 2016. In order to account for variability in time at given locations, similar sampling sites to those described in Nasrabadi et al. [18] were chosen for the present study. Water samples were taken from the main channels at a minimum distance of 1 m from the river banks and 15 cm below the water surface to avoid any disturbance by floating debris or local bank erosion. Water movement was fast enough to allow for well mixed and turbid samples [24]. Samples were collected in pre-washed and rinsed 500 ml HDPE bottles. To cover a larger range of TSS values, additional high-turbidity samples were produced in the laboratory by mixing and re-suspending fine-grained river bed sediments in river water (grab samples taken from the respective location) in 10 liter HDPE containers. A schedule was designed to sample the supernatant water after distinct time periods as described by Ruegner et al. [16]. Using this approach, six composite samples covering a broad TSS range were generated. These artificial samples were treated in the same way as the natural samples.

**Fig 2:**
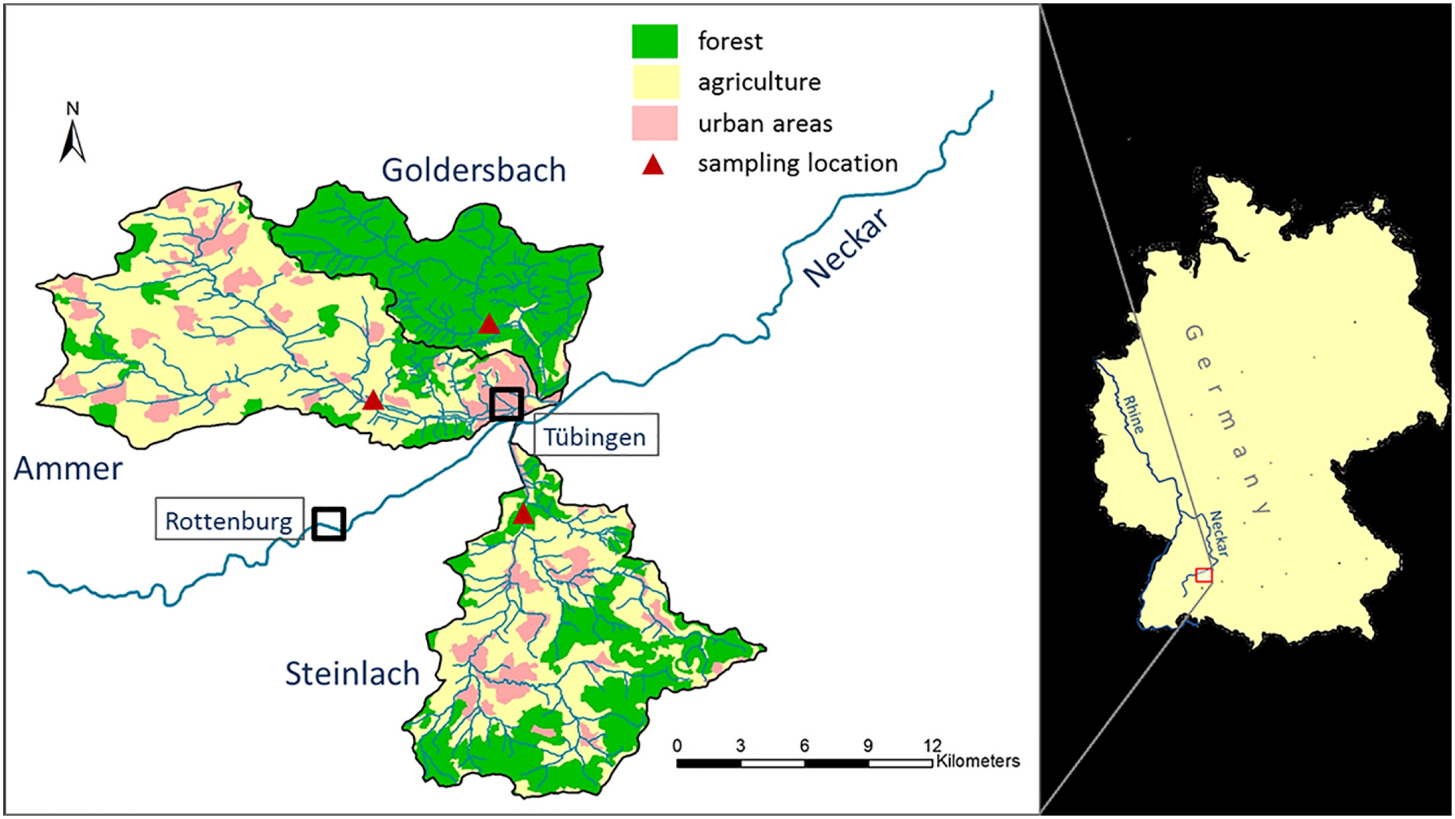
Map of the Ammer, Goldersbach, and Steinlach catchments in Southwest Germany with major types of land-use as well as the sampling locations. Reprinted from [11] under a CC BY license, with permission from Elsevier, original copyright 2013.

The **Ammer River** is a 5^th^-order tributary of the Neckar River in southwestern Germany and one of the principal tributaries of the Rhine (Fig 2). The length of the main stem is 22 km and it drains a total area of 238 km^2^. The geology is dominated by Triassic limestones and gypsum-bearing layers that are partly karstified, as well as mudstones and, along the valleys floors, alluvial clays. The relief of the sampled catchment ranges from 346 to 600 m asl. Land use is mostly agriculture (71%) while >17% of the catchment is covered by urban areas (Fig 2) and 12% by forest. The mean discharge at the gauging station at Pfäffingen is 1 m^3^/s. Event-related high discharge samples were taken at a gauging station which monitors the upper 134 km^2^ of the catchment in July 2013 and May 2014 using an automated sampler. Samples were collected in high density polyethylene (HDPE) bottles.

The **Goldersbach River** is a 4^th^-order tributary of the Ammer River, draining an area of 73 km^2^ with altitudes varying between 320 and 580 m asl. The main stem has a length of 18.7 km and is fed by a stream network of 130 km total length. The catchment is dominated by a plateau of Middle and Upper Triassic sandstones with valleys cutting into the underlying marlstones and, only locally, into gypsum-bearing mudstones. The gauged part of the catchment has an area of 37.6 km^2^, is completely forested and part of a nature reserve; thus, human impacts are low. The mean discharge at the gauge is 0.25 m^3^ s^-1^. Flood-event samples were taken by automated sampling at this gauging station in July 2013 and transferred to HDPE bottles.

The **Steinlach River**, a 4^th^-order tributary of the Neckar River, has a total length of 25 km, whereas the length of its stream network is about 190 km. The Steinlach drains a total catchment area of 140 km^2^ with a mean discharge of 1.7 m^3^/s. Elevation ranges between 320 and 880 m asl. The catchment geology comprises three principle sedimentary rock formations: Upper Jurassic (mainly limestones), covering the upstream parts of the catchment; Middle Jurassic (dominated by mudstones, marls and sandstones), covering the central portion of the catchment and Lower Jurassic (made up by black shales, mudstones and carbonates), covering the lower parts of the catchment. Upper Triassic rocks (sandstones and marls/mudstones) are only present near the confluence with the Neckar River. These formations dip approximately 1–2° in the direction ESE [20]. Land use within the study catchment comprises 49% agricultural areas, 39% forests and semi-natural areas and 12% artificial surfaces. Event-related high discharge samples with intermediate to high turbidities (i.e. suspended particle concentrations) were collected in May as well as early and late July 2014 at a monitoring station approximately 4 km upstream of the Steinlach River confluence with the Neckar River (Fig 2) and transferred in HDPE bottles.

### Laboratory treatment

The samples from the Haraz catchment were analyzed using the US EPA method 200.2 [25] for the determination of total element concentrations. In brief, a mixture of concentrated (70%) nitric acid and concentrated (40%) hydrochloric acid (1.0 ± 0.1 ml conc. HNO_3_ and 0.50 ± 0.05 ml conc. HCl) was used as the extracting agent. Well-homogenized 50 mL sub-samples were then digested for 2 to 2.5 hours at 95° ± 5°C. Quality assurance/quality control procedures were applied according to the standards cited. Filtered digested samples were then measured using inductively coupled plasma atomic emission spectrometry (ICP-AES) according to the EPA-3005 method [26]. Accuracy was also cross-checked by the determination of standards and random duplicates concentrations. Deviation from target values was less than ±5% for each element. For determination of TSS values EPA method 160.2 was used [27].

Total element concentrations (bulk water samples) in the Steinlach, Ammer and Goldersbach samples were analyzed according to the German standard DIN EN ISO 11885 [28]. In brief, 20 ml of the water sample, including suspended sediments, is digested using 2 ml HNO_3_ plus 0.5 ml H_2_O_2_ and a microwave extraction. Metals were analyzed using ICP-MS/ICP OES equipment. TSS was determined by filtration (Whatman 934-AH Glass Microfiber filters, 1.5 μm), with subsequent drying at 105°C for 24 h and recording of the mass of the dried filter cakes (according to the standard procedure DIN 38402 A 24 [29].

## Results and discussion

The dissolved (*C*_*W*_, intercepts) and particle-bound (*C*_*SUS*_, slopes) concentrations of single metals were calculated for each event/sampling campaign using linear regressions between total concentrations of metals and the respective TSS values in river water/artificial suspensions (according to Eq 1).

### Lower Haraz Basin

**Fig 3:**
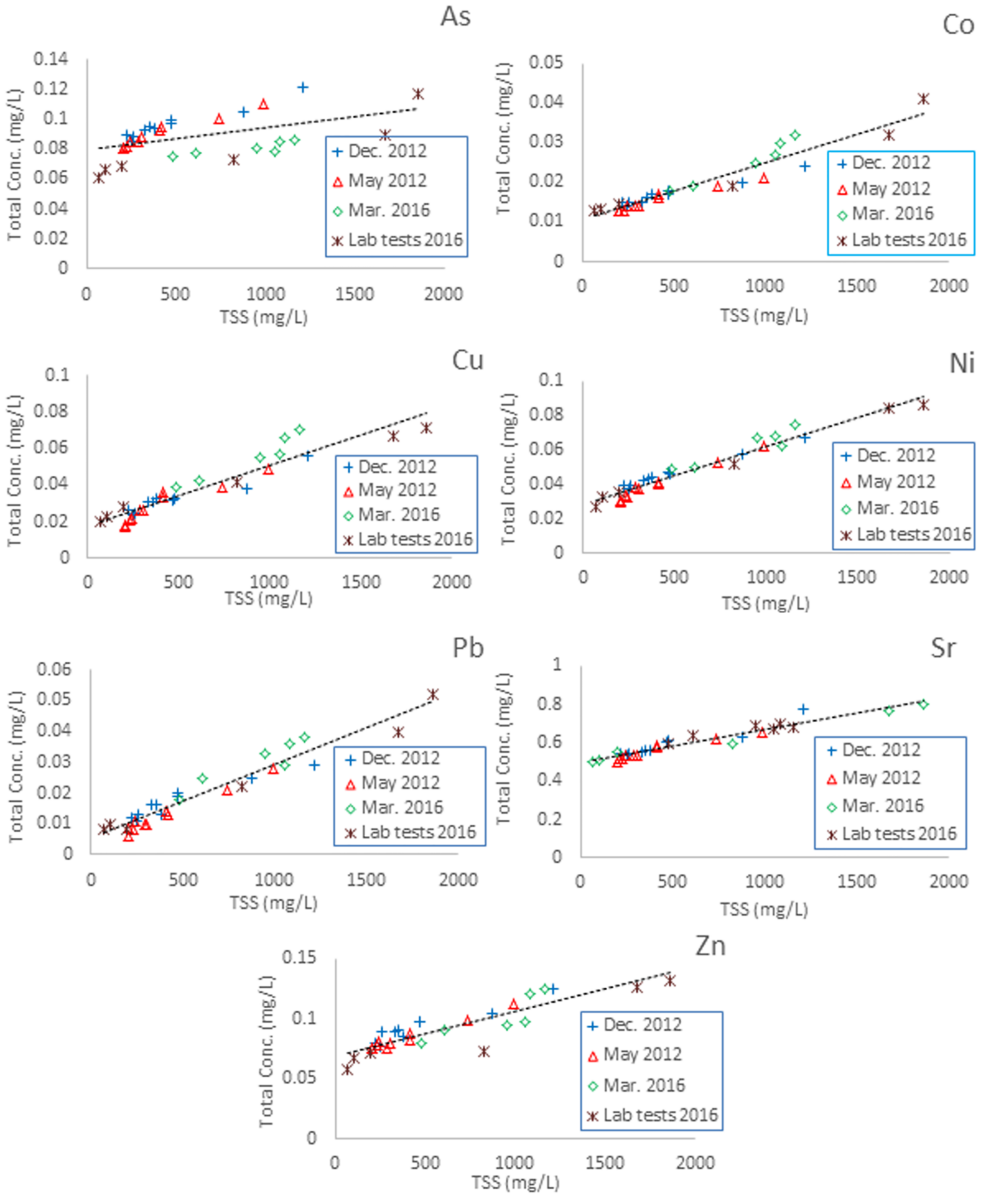
Linear regressions of total element concentrations and TSS during different sampling campaigns in the lower Haraz catchment (March 2016 plus lab tests), 2012 data adapted from Nasrabadi et al. [18].

These data cover a TSS range of 481–1161 mg L^-1^ (March 2016) and are corroborated with a laboratory test containing suspended sediments in the range of 66–1860 mg L^-1^. Robust linear correlations of *C*_*W*,*tot*_ and TSS are observed (see Fig 3) for all elements displaying distinct values for *C*_*SUS*_ and pronounced intercepts (*C*_*W*_). The additional laboratory tests–although spanning a larger TSS range–show very good agreement with the natural bulk river water samples. Both data sets were also in close agreement with data reported by Nasrabadi et al. [18] for samples taken from ten locations distributed over the whole Haraz Catchment in May and December 2012 (also displayed in S1 Table). This indicates that the variability of *C*_*SUS*_ and *C*_*W*_ is low in the river water despite the relatively large catchment area and diverse geology. One reason for the low variability may be widespread sand and gravel mining activities taking place in the Haraz River [18]. The influence of such a homogenization was also observed by Ji et al. in the Baihe and Chaohe Rivers in China, which are heavily exposed to gold and iron mining activities [30]. They also reported relatively low coefficients of variation (CV) for particle-bound concentrations of different metals within the frame of a spatially and temporally variable dataset. Overall, correlations (all data) thus show only little variability between different campaigns except for arsenic–this could be due to the influence of hydrothermal springs in the catchment [31].

A comparison between *C*_*SUS*_ values obtained in the present study and concentrations in the bed sediment measured at the same locations of the Haraz basin in August 2007 [23] (for a grain size fraction < 63 μm) shows good agreement for elements Cu, Co, Pb and Ni (less than 20% of difference). For As, Sr and Zn, however, differences are observed which may be due to heterogeneities in the river bed sediment or different grain size distributions of suspended particles and river bed sediments [18]. In particular, fine particles (< 63 mm) in water usually tend to additionally adsorb metals [3,32]. Grain size distributions in suspended sediments depend on flow velocity and thus the magnitude of the discharge event. However, in the southwestern German catchments, mean grain diameters of suspended sediments sampled during high flow events with suspended particle concentrations between 70 and 2500 mg L^-1^ were determined to range from 15–35 μm and thus are very close to the grain size distribution observed in river sediments for the fraction < 63 μm [16].

### Ammer catchment

**Fig 4:**
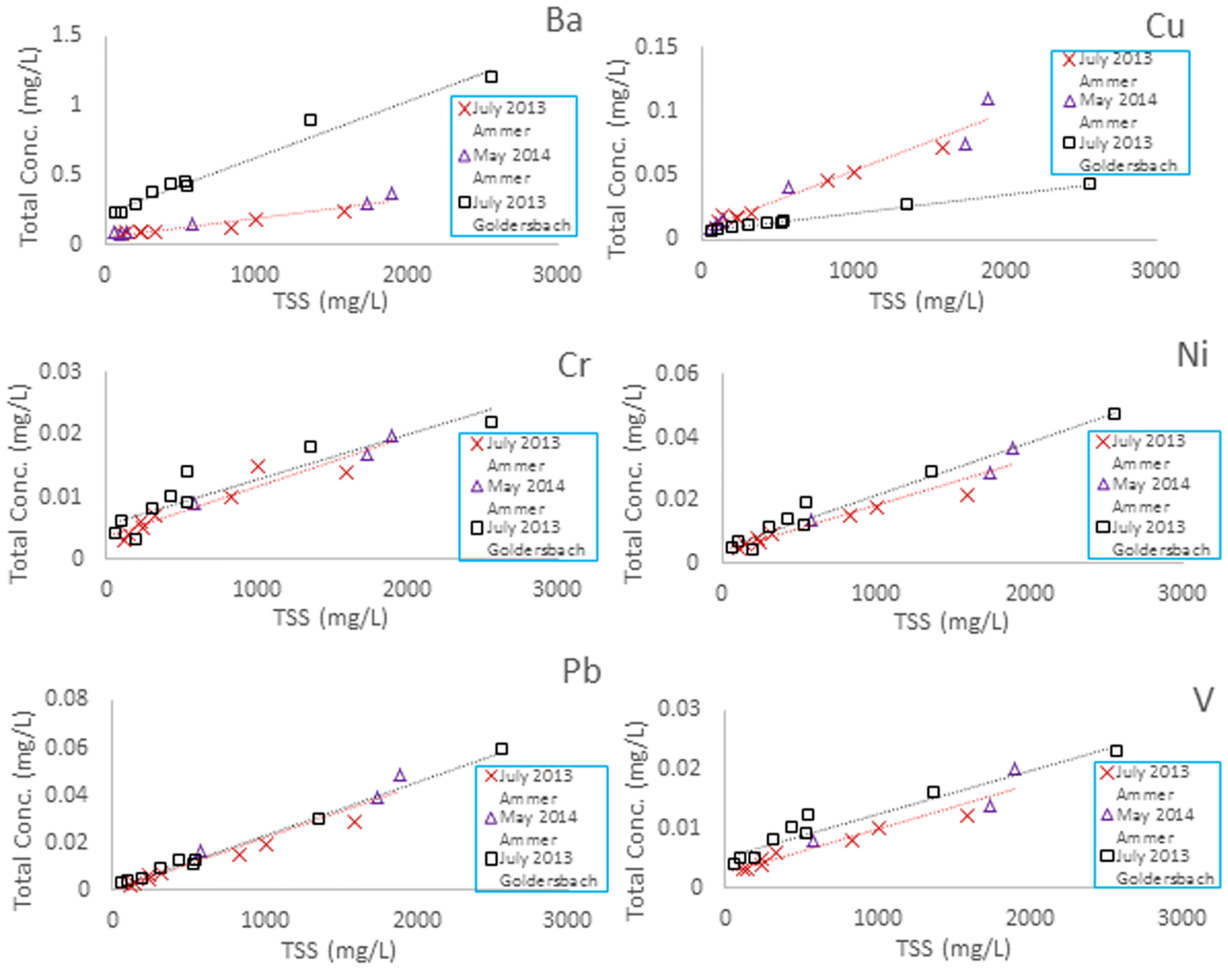
Linear regressions of total element concentrations and TSS during the 2013 and 2014 sampling campaigns in the Ammer and Goldersbach catchments, Germany.

Here, a TSS range of 112–1590 mg L^-1^ (July 2013) and 55–1891 mg L^-1^ (May 2014) was covered by a sampling campaign at a single measurement location. Robust linear correlations of *C*_*W*,*tot*_ and TSS are observed for all selected elements (see Fig 4) during two independent high discharge events (separated by almost one year). The concentrations of Pb, Cu, Ni, and Zn on suspended solids are in the geogenic background ranges for soils/subsoils in the State of Baden-Württemberg, according to the data reported by LABO [33] and Lotze [34]. Only Cu and Zn are slightly above and Cr below the average background values. Except for lead, clear intercepts (*C*_*W*_) are detected. Both data sets were in close agreement which suggests only little temporal variability of *C*_*SUS*_ and *C*_*W*_ between the sampled events.

### Goldersbach catchment

A TSS range of 68–2562 mg L^-1^ was covered during the sampling campaign in July 2013. Mostly good correlations are observed for Cr and V. The slopes and intercepts are strikingly similar to the Ammer results with two exceptions: Dissolved and particulate concentrations of Ba are generally higher in the Goldersbach catchment, which is probably is due to a feldspar bearing sandstone formation occurring there. At the same time, Cu concentrations that are clearly below those in the other three catchments might be related to missing anthropogenic inputs into the forested Goldersbach catchment (see Fig 4).

### Steinlach catchment

**Fig 5:**
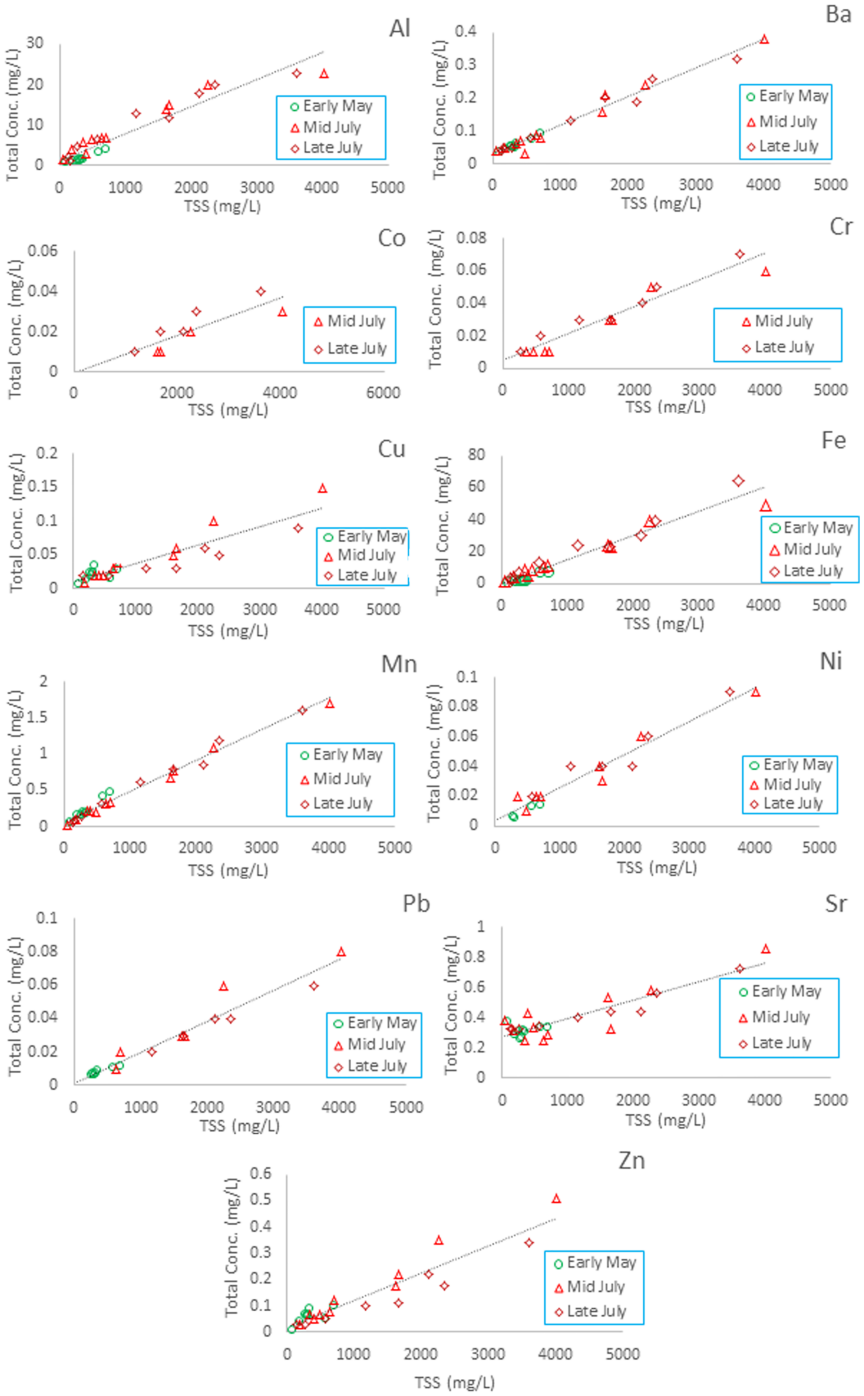
Linear regressions of total element concentrations and TSS during the 2014 sampling campaigns in the Steinlach catchment, Germany.

A TSS range of 85–703 mg L^-1^ (May 2014), 46–4016 mg L^-1^ (mid-July 2014) and 129–3615 mg L^-1^ (Late-July 2014) was covered. Water samples taken at a single location at three independent events show reasonable good linear correlations of *C*_*W*,*tot*_ and TSS for most elements (see Fig 5). For some data sets (Sr, Cu, and Zn) correlations were slightly weaker. For some metals (e.g. Co), intercepts (*C*_*W*_) are uncertain (close or below zero) which could be due to the limited number of data in the low concentration range, but could also indicate that dissolved concentrations are indeed very low. Despite this, all data sets–and here in particular data for *C*_*SUS*_—show only little variability and differ only slightly from overall linear correlations. Particle-bound concentrations of As, Pb, Cr, Cu, Ni and Zn may be attributed to the catchment geology [33,34]. However, as in the Ammer catchment, Cu and Zn are slightly above the average. The slightly higher Cr concentrations in suspended sediments in comparison to the neighboring Ammer and Goldersbach catchments might be explained by contributions from widespread Jurassic limestones [33].

By comparing results across all four study areas it can be concluded that in the Haraz catchment dissolved concentrations tend to be higher (e.g., Co, Ni, Pb). A possible explanation is the active volcanism in the Haraz catchment and the associated hydrothermal springs. Unlike in the southwest German catchments in the present study, the suspended particle concentrations are high also during low flow conditions due to the mining activities along the river channel. Consequently, the fraction of highly mineralized water from hydrothermal springs is always relatively high and therefore even turbid water shows relatively high dissolved metal concentrations (large intercepts, Fig 3). In contrast, the particle-bound concentrations are surprisingly close to the Ammer-, Steinlach- and Goldersbach catchments (e.g., Ni, Pb). This could be due to the similar geology, e.g. the occurrence of sedimentary rocks such as limestones and mudstones. Particle-bound and dissolved concentrations as well as statistical details of linear regressions for all rivers are displayed in S1 Table.

### Metals distribution in the solid and liquid phase

**Fig 6:**
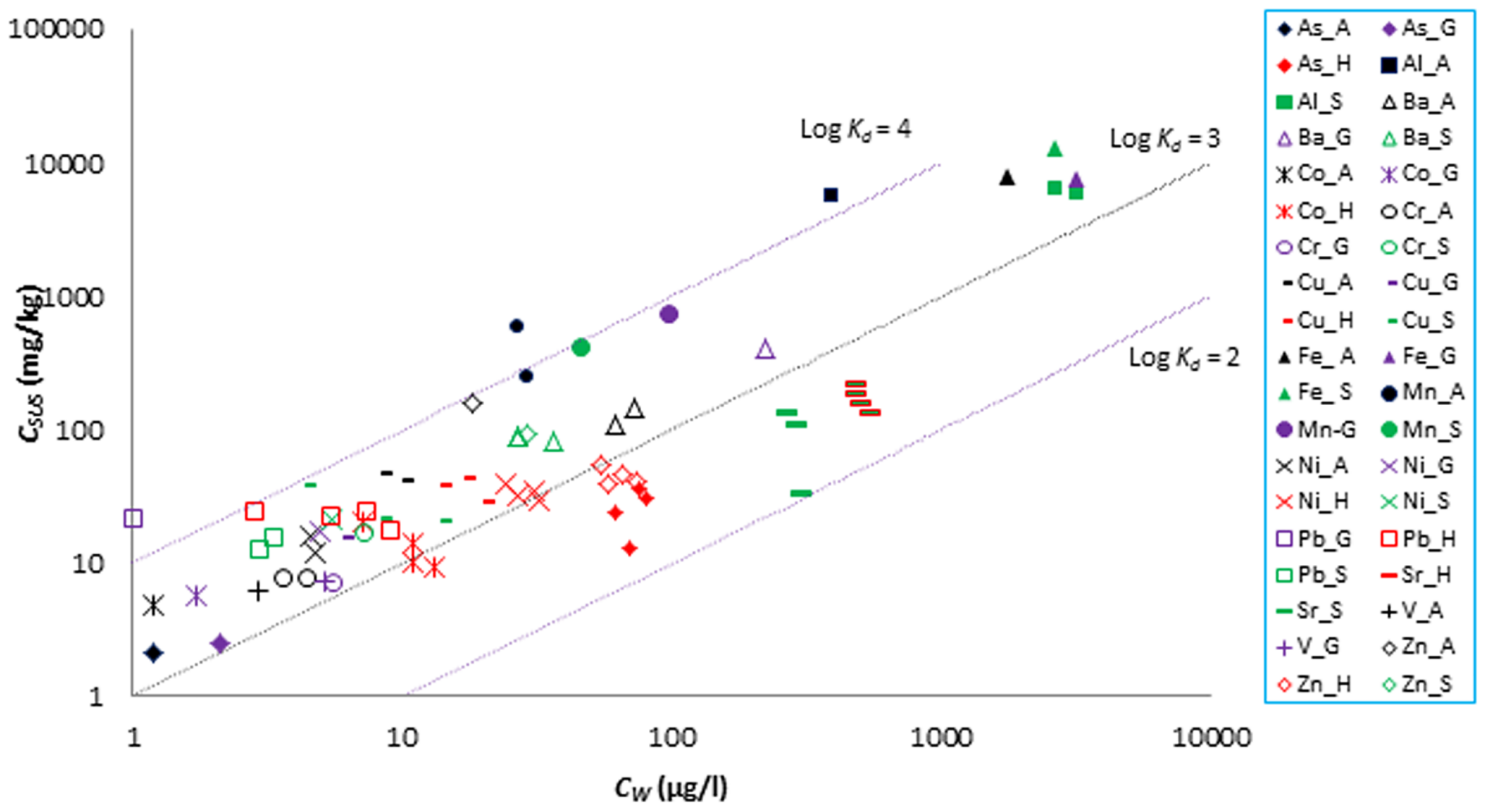
Comparison of particulate *(C*_*SUS*_) and dissolved *(C*_*W*_) concentrations of metals in all four catchments [Ammer (_A, in black), Haraz (_H, in red), Steinlach (_S, in green) and Goldersbach (_G, in purple)].

The scatter plot of *C*_*SUS*_ vs. *C*_*W*_ (Fig 6) for all elements in different catchments generally shows a good correlation between dissolved and particulate concentrations for metals across most catchments with the exception of the Haraz values. Generally, calculated *C*_*SUS*_/*C*_*W*_ values may be interpreted as distribution coefficients *(K*_*d*_) between water and sediment, and for most metals in all four study catchments these values indicate a strong affinity to suspended particles. *K*_*d*_ values in this study are typically between 1000 and 10 000 L kg^-1^. Low *K*_*d*_ values may, in a first instance, be considered as an indication towards elevated bioavailability and higher risk level. In the Haraz Basin, for example, remarkably low log *K*_*d*_ values (average log *C*_*SUS*_/*C*_*W*_ = 2.6 L kg^-1^) are observed for arsenic in comparison with other studies, such as 3.4 L kg^-1^ in the Taehwa River, South Korea [35], 4.6 L kg^-1^ in East-Hainan estuaries, China [36], 3.8 L kg^-1^ in the Seine estuary, France [37], and 3.8 L kg^-1^ in the Paranagua estuary, Brazil [38]. In the Haraz Basin, hot springs as well as coal-rich formations may be the source of high concentrations of dissolved arsenic in the river. Higher arsenic concentrations in the dissolved fraction, and the accumulation of arsenic in biota (e.g. rainbow trout) have been thoroughly discussed in the literature [39,40]. A relatively weak affinity to solid phases was also observed for Sr in both the Haraz and the Steinlach catchment (average log *C*_*SUS*_/*C*_*W*_ = 2.5 to 2.6 L kg^-1^). This was reported also by Ji et al. [30] for Sr as an indicator element of soil and rock weathering, and by Beltaos and Burrel [8] with similarly low log *C*_*SUS*_/*C*_*W*_ values (2.7 L kg^-1^) in comparison to other metals. The Haraz basin also shows lower *K*_*d*_ for Cu, Ni, Pb and Zn in comparison with the German (Ammer and Steinlach) study catchments.

### Comparison to background values and data from literature

In Table 1 we compare the average concentrations of metals on suspended solids within the investigated catchments with data from background soils/regional sediments. In general, a good agreement for Pb, Ni, Cr, Co, and also Cu and Zn is observed which indicates the predominance of geogenic influence. However, Cu and Zn–often associated with anthropogenic pollution from urban areas [41]–are elevated in particular in the Ammer and Steinlach catchments. Polycyclic aromatic hydrocarbons (PAHs) are also particularly elevated in these catchments, indicating distinct urban influences [16,17]. Nevertheless, heavy metals in the Ammer catchment are surprisingly low compared to significant pollution by polychlorinated biphenyls (in fish) and PAH in (suspended) sediments [11]. This indicates that metal fluxes from urban areas are much less pronounced compared to the geogenic background; this contrasts to fluxes of persistent organic pollutants for which cities are hot spots.

**Table 1:** A comparison among particle-bound concentration of metals (mg/kg) in Ammer, Steinlach, Goldersbach and Haraz River (calculated in this study) with similar studies/ background values.

Region	Cu	Pb	Ni	Cr	Zn	Sr	Co
Haraz River*	33	24	34	-	37	172	14
Ammer River*	45	23	15	8.2	160**	-	4.9**
Steinlach River*	28	19	23	16	104	122	9.4
Goldersbach River**	15	22	17	7.3	-	-	5.8
**Data from literature / background values**							
Haraz River Estuary, Iran [23]	32	26	44	28	74	603	10
Average soils on arable land in Baden-Württemberg [33]	19	27	27	36	60	-	-
Average soils on arable land in alluvial floodplains in Germany	14	29	15	15	50	-	-
Upper continental crust [50]	28	17	47	92	67	320	17
Yellow River delta, China [51]	21	21	27	62	61	-	-
Yangtze River Estuary, China [52]	25	24	-	72	83	-	-
Brisbane River estuary, Australia [53]	29	26	15	15	107	64	15
Buyak Menderes River, Turkey [42]	137	54	315	165	120	-	29
St. Lawrence River harbor, Canada [6]	108	58	43	69	306	-	-

* Data from this study

**Extracted from single datasets

The role of anthropogenic sources with respect to increased metal concentrations in natural soils/sediments is delineated by many researchers: Relatively high concentrations of metals were detected in the Buyak Menderes, Turkey [42] and in the St. Lawrence River harbor, Canada [6]. In Turkey these were attributed to intensive discharge of industrial wastewater and in Canada to heavy waterway traffic as well as urban loads.

Dissolved concentrations of metals investigated in the present study are higher than the world average and also higher than in other well-known large rivers [43]. A better agreement is observed when dissolved concentrations in this study are compared to those from urban runoff [44,45]. Generally, dissolved concentrations of metals in basins fed by intensified urban/agricultural land use [46–48] are higher (sometimes up to three orders of magnitude) than those in natural areas [44,49].

### Implications

Our study demonstrates that robust linear relationships between TSS and total metal concentrations exist in rivers of four contrasting catchments and which are stable over time (e.g. independent on season and events). Rivers are thus “integrators” in catchments and give a representative measure of pollutant fluxes coming from upstream areas. The method implemented in this study not only offers a robust way to measure pollutants fluxes but it also allows particle-bound and dissolved metal fluxes to be distinguished. For instance, a mean annual TSS of more than 100 mg l^-1^ at *K*_*d*_ (= *C*_*SUS*_*/C*_*W*_) values of 10000 l kg^-1^ (which is typical for the study catchments) indicates the shift from dissolved- to particle- dominated metal fluxes. As the method is based on suspended sediments, it is less affected by local heterogeneities in comparison to conventional sediment sampling schemes that are based on grab samples from the local river bed. The method also proved to be cost-efficient in comparison to standard metal analyses, which generally include both bulk and speciation procedures. The regression-based method also allows the risk level of freely dissolved metals concentrations to be evaluated by utilizing *the intercept (C*_*W*_) values: the higher *C*_*W*_, the higher the potential risk level assigned to biota, as demonstrated for arsenic in the Haraz, where concentrations in river water were reported to be up to ten times higher than WHO guidelines [39]. The results of the present study show that relationships between bulk metal concentrations and total suspended solids in rivers are catchment-specific and time-invariant–and that such methods can be extended to other field sites. In principle, the methods used in this study could also be applied to other compounds such as polychlorinated biphenyls, brominated flame retardants[12] or elements including radionuclides (^210^Pb, ^137^Cs, ^7^Be), potentially allowing the origin and age of suspended sediments in rivers to be traced [54,55].

## Supporting information

S1 File Granted permission for Fig 1. (PDF)

S2 File Granted permission for Fig 2. (PDF)

S1 Table Results from linear regressions [C_W_: Dissolved concentration of elements, C_SUS_: Particle-bound concentrations]. (DOCX)
