# Supplementary material for: Bulk metal concentrations versus total suspended solids in rivers: Time-invariant & catchment-specific relationships
Source: PLoS One. 2018 Jan 17;13(1):e0191314. doi: 10.1371/journal.pone.0191314 (PMC5771599; doi:10.1371/journal.pone.0191314)
Supplement: S1 Table — (DOCX) [file pone.0191314.s003.docx]

S1 Table. Results from linear regressions [C_W_: dissolved concentration of elements, C_SUS_: particle-bound concentrations].

| Metal/ Metalloid | *Sample* | C_W_ (µg/l) | C_SUS_ (mg/kg) | Log *C_SU_*_S_/*C_W_* (L/kg) | N | R^2^ |
| --- | --- | --- | --- | --- | --- | --- |
| As | Haraz March 2016 | 69 ± 3.5 | 13 ± 3.8 | 2.3 | 6 | 0.75 |
|  | Haraz lab tests | 61 ± 5.4 | 24 ± 5.0 | 2.6 | 6 | 0.85 |
|  | Haraz May 2012* | 76 ± 1.4 | 36 ± 3.0 | 2.7 | 10 | 0.95 |
|  | Haraz Dez 2012* | 81 ± 1.5 | 31 ± 2.7 | 2.6 | 10 | 0.94 |
|  | *Haraz all data* | *80 ± 3.5* | *15 ± 4.7* | *2.3* | *32* | *0.26* |
|  | Ammer July 2013 | 1.2 ± 0.3 | 2.1 ± 0.3 | 3.2 | 8 | 0.86 |
|  | Goldersbach July 2013 | 2.1 ± 0.4 | 2.5 ± 0.4 | 3.1 | 9 | 0.84 |
| Al | Ammer May 2014 | 394 ± 188 | 5540 ± 175 | 4.1 | 6 | 1.0 |
|  | Steinlach May 2014 | (≤ 0) | 5760 ± 650 | -- | 9 | 0.91 |
|  | Steinlach July 2014a | 3180 ± 941 | 5775 ± 590 | 3.3 | 11 | 0.92 |
|  | Steinlach July 2014b | 2625 ± 1130 | 6420 ± 645 | 3.4 | 9 | 0.93 |
|  | *Steinlach all data* | *1420 ± 570* | *6640 ± 405* | *3.7* | *29* | *0.91* |
| Ba | Ammer July 2013 | 62 ± 7.8 | 107 ± 10 | 3.2 | 8 | 0.94 |
|  | Ammer May 2014 | 72 ± 10 | 145 ± 9.4 | 3.3 | 6 | 0.98 |
|  | *Ammer all data* | *60 ± 10* | *135 ± 11* | *3.4* | *14* | *0.93* |
|  | Goldersbach July 2013 | 223 ± 25 | 405 ± 24 | 3.3 | 9 | 0.98 |
|  | Steinlach May 2014 | 27 ± 2.8 | 89 ± 7.3 | 3.5 | 9 | 0.95 |
|  | Steinlach July 2014a | 27 ± 8.1 | 90 ± 5.1 | 3.5 | 11 | 0.97 |
|  | Steinlach July 2014b | 36 ± 10 | 83 ± 5.5 | 3.4 | 9 | 0.97 |
|  | *Steinlach all data* | *29 ± 3.8* | *88 ± 2.7* | *3.5* | *29* | *0.97* |
| Co | Haraz March 2016 | 7.2 ± 2.1 | 20 ± 2.3 | 3.4 | 6 | 0.95 |
|  | Haraz lab tests | 11 ± 1.8 | 14 ± 1.7 | 3.1 | 6 | 0.95 |
|  | Haraz May 2012* | 11 ± 0.40 | 10 ± 0.84 | 3.0 | 10 | 0.95 |
|  | Haraz Dez 2012* | 13 ± 0.41 | 9.2 ± 0.72 | 2.9 | 10 | 0.95 |
|  | *Haraz all data* | *11 ± 0.6* | *14 ± 0.8* | *3.1* | *32* | *0.91* |
|  | Ammer July 2013 | 1.2 ± 0.1 | 4.9 ± 0.2 | 3.6 | 8 | 0.99 |
|  | Goldersbach July 2013 | 1.7 ± 0.4 | 5.8 ± 0.4 | 3.5 | 9 | 0.97 |
|  | Steinlach July 2014a | (≤ 0) | 8.2 ± 1.5 | -- | 4 | 0.93 |
|  | Steinlach July 2014b | (≤ 0) | 12 ± 1.9 | -- | 5 | 0.93 |
|  | *Steinlach all data* | (≤ 0) | *9.4 ± 2.2* | *--* | *9* | *0.73* |
| Cr | Ammer July 2013 | 3.6 ± 1.0 | 7.9 ± 1.3 | 3.3 | 8 | 0.85 |
|  | Ammer May 2014 | 4.4 ± 1.9 | 7.8 ± 1.2 | 3.3 | 3 | 0.97 |
|  | *Ammer all data* | *3.6 ± 0.8* | *8.2 ± 0.8* | *3.4* | *11* | *0.93* |
|  | Goldersbach July 2013 | 5.5 ± 1.2 | 7.3 ± 1.2 | 3.1 | 9 | 0.84 |
|  | Steinlach July 2014a | 1.5 ± 2.5 | 16 ± 1.5 | 4.0 | 8 | 0.94 |
|  | Steinlach July 2014b | 7.1 ± 2.7 | 17 ± 1.3 | 3.4 | 7 | 0.97 |
|  | *Steinlach all data* | *5.0 ± 2.5* | *16 ± 1.3* | *3.5* | *15* | *0.92* |
| Cu | Haraz March 2016 | 17 ± 5.3 | 42 ± 5.8 | 3.4 | 6 | 0.93 |
|  | Haraz lab tests | 20 ± 1.0 | 28 ± 0.9 | 3.1 | 6 | 1.00 |
|  | Haraz May 2012* | 14 ± 2.3 | 37 ± 4.8 | 3.4 | 10 | 0.88 |
|  | Haraz Dez 2012* | 20 ± 1.7 | 28 ± 3.1 | 3.1 | 10 | 0.91 |
|  | *Haraz all data* | *18 ± 1.6* | *33 ± 2.1* | *3.3* | *32* | *0.89* |
|  | Ammer July 2013 | 10 ± 1.4 | 40 ± 2.0 | 3.6 | 8 | 0.98 |
|  | Ammer May 2014 | 8.4 ± 5.9 | 47 ± 5.5 | 3.7 | 6 | 0.95 |
|  | *Ammer all data* | *8.4 ± 2.6* | *45 ± 2.9* | *3.7* | *14* | *0.95* |
|  | Goldersbach July 2013 | 6.0 ± 0.3 | 15 ± 0.3 | 3.4 | 9 | 1.0 |
|  | Steinlach May 2014 | 14 ± 5.6 | 20 ± 14 | 3.2 | 9 | 0.21 |
|  | Steinlach July 2014a | 4.4 ± 3.3 | 37 ± 2.0 | 3.9 | 10 | 0.97 |
|  | Steinlach July 2014b | 8.4 ± 6.1 | 21 ± 3.1 | 3.4 | 7 | 0.90 |
|  | *Steinlach all data* | *9.4 ± 3.6* | *28 ± 2.5* | *3.5* | *26* | *0.84* |
| Fe | Ammer July 2013 | 1760 ± 634 | 7770 ± 848 | 3.6 | 8 | 0.93 |
|  | Ammer May 2014 | 151 ± 153 | 9870 ± 142 | 4.8 | 6 | 1.0 |
|  | *Ammer all data* | *899 ± 491* | *9090 ± 464* | *4.0* | *14* | *0.97* |
|  | Goldersbach July 2013 | 3160 ± 620 | 7340 ± 610 | 3.4 | 9 | 0.96 |
|  | Steinlach May 2014 | (≤ 0) | 12140 ± 1400 | -- | 9 | 0.91 |
|  | Steinlach July 2014a | 2640 ± 1380 | 12600 ± 860 | 3.7 | 11 | 0.96 |
|  | Steinlach July 2014b | 817 ± 1940 | 16400 ± 1100 | 4.3 | 9 | 0.97 |
|  | *Steinlach all data* | *270 ± 970* | 15000 ± 700 | 4.8 | *29* | *0.95* |
| Mn | Ammer July 2013 | 29 ± 22 | 253 ± 29 | 3.9 | 8 | 0.93 |
|  | Ammer May 2014 | 27 ± 22 | 593 ± 21 | 4.3 | 6 | 1.0 |
|  | *Ammer all data* | (≤ 0) | 497 ± 68 | -- | 14 | 0.82 |
|  | Goldersbach July 2013 | 96 ± 45 | 738 ± 44 | 3.9 | 9 | 0.98 |
|  | Steinlach May 2014 | (≤ 0) | 672 ± 51 | -- | 9 | 0.96 |
|  | Steinlach May 2014 | (≤ 0) | 672 ± 51 | -- | 9 | 0.96 |
|  | Steinlach July 2014a | 45 ± 20 | 424 ± 12 | 4.0 | 11 | 0.99 |
|  | Steinlach July 2014b | 38 ± 39 | 443 ± 22 | 4.1 | 9 | 0.98 |
|  | *Steinlach all data* | *58 ± 15* | *429 ± 11* | *3.5* | *29* | *0.98* |
| Ni | Haraz March 2016 | 31 ± 6.4 | 35 ± 6.9 | 3.1 | 6 | 0.86 |
|  | Haraz lab tests | 27 ± 1.5 | 32 ± 1.3 | 3.1 | 6 | 0.99 |
|  | Haraz May 2012* | 24 ± 0.83 | 39 ± 1.8 | 3.2 | 10 | 0.98 |
|  | Haraz Dez 2012* | 32 ± 0.64 | 29 ± 1.1 | 3.0 | 10 | 0.98 |
|  | *Haraz all data* | *28 ± 1.0* | *34 ± 1.3* | *3.1* | *32* | *0.96* |
|  | Ammer July 2013 | 4.7 ± 0.6 | 12 ± 0.74 | 3.4 | 8 | 0.97 |
|  | Ammer May 2014 | 4.6 ± 5.9 | 16 ± 3.9 | 3.5 | 3 | 0.94 |
|  | *Ammer all data* | *3.7 ± 1.2* | *15 ± 1.2* | *3.6* | *11* | *0.94* |
|  | Goldersbach July 2013 | 4.9 ± 1.2 | 17 ± 1.2 | 3.5 | 9 | 0.97 |
|  | Steinlach May 2014 | (≤ 0) | 22 ± 3.5 | -- | 4 | 0.95 |
|  | Steinlach July 2014a | 5.5 ± 3.6 | 21 ± 1.9 | 3.6 | 8 | 0.95 |
|  | Steinlach July 2014b | 7.0 ± 7.3 | 22 ± 3.4 | 3.5 | 6 | 0.91 |
|  | *Steinlach all data* | *2.9 ± 2.4* | *23 ± 1.4* | *3.9* | *18* | *0.95* |
| Pb | Haraz March 2016 | 7.3 ± 4.6 | 25 ± 5.0 | 3.5 | 6 | 0.86 |
|  | Haraz lab tests | 5.4 ± 2.0 | 23 ± 1.8 | 3.6 | 6 | 0.97 |
|  | Haraz May 2012* | 2.8 ± 0.69 | 25 ± 1.5 | 3.9 | 10 | 0.97 |
|  | Haraz Dez 2012* | 8.9 ± 1.1 | 18 ± 2.0 | 3.3 | 10 | 0.91 |
|  | *Haraz all data* | *5.3 ± 0.9* | *24 ± 1.2* | *3.7* | *32* | *0.93* |
|  | Ammer July 2013 | (≤ 0) | 18 ± 0.44 | -- | 8 | 0.99 |
|  | Ammer May 2014 | 3.7 ± 6.9 | 22 ± 4.6 | 3.8 | 3 | 0.95 |
|  | *Ammer all data* | (≤ 0) | *23 ± 1.8* | *--* | *11* | *0.95* |
|  | Goldersbach July 2013 | 0.9 ± 0.6 | 22 ± 0.6 | 4.4 | 9 | 1.0 |
|  | Steinlach May 2014 | 2.9 ± 0.9 | 13 ± 2.1 | 3.7 | 7 | 0.88 |
|  | Steinlach July 2014a | 1.3 ± 6.1 | 20 ± 2.8 | 4.2 | 6 | 0.93 |
|  | Steinlach July 2014b | 3.3 ± 2.7 | 16 ± 1.1 | 3.7 | 5 | 0.98 |
|  | *Steinlach all data* | *1.1* ± *2.0* | *19 ± 1.1* | *4.2* | *18* | *0.94* |
| Sr | Haraz March 2016 | 543 ± 26 | 133 ± 27 | 2.4 | 6 | 0.85 |
|  | Haraz lab tests | 497 ± 12 | 158 ± 11 | 2.5 | 6 | 0.98 |
|  | Haraz May 2012* | 484 ± 9.3 | 186 ± 20 | 2.6 | 10 | 0.91 |
|  | Haraz Dez 2012* | 485 ± 13 | 222 ± 23 | 2.7 | 10 | 0.92 |
|  | *Haraz all data* | *499 ± 6.7* | *172 ± 9.0* | *2.5* | *32* | *0.92* |
|  | Steinlach May 2014 | 301 ± 27 | 33 ± 69 | 2.0 | 9 | 0.03 |
|  | Steinlach July 2014a | 264 ± 39 | 135 ± 24 | 2.7 | 11 | 0.77 |
|  | Steinlach July 2014b | 291 ± 21 | 107 ± 12 | 2.6 | 9 | 0.92 |
|  | *Steinlach all data* | *274 ± 16* | *122 ± 11* | *2.6* | *29* | *0.82* |
| V | Ammer July 2013 | 2.9 ± 0.44 | 6.1 ± 0.59 | 3.3 | 8 | 0.94 |
|  | Ammer May 2014 | 3.3 ± 5.1 | 7.6 ± 3.4 | 3.4 | 3 | 0.83 |
|  | *Ammer all data* | *2.6 ± 0.8* | *7.5 ± 0.8* | *3.5* | *11* | *0.92* |
|  | Goldersbach July 2013 | 5.2 ± 0.7 | 7.4 ± 0.7 | 3.2 | 9 | 0.94 |
| Zn | Haraz March 2016 | 54 ± 16 | 54 ± 17 | 3.0 | 6 | 0.71 |
|  | Haraz lab tests | 58 ± 5.8 | 39 ± .3 | 2.8 | 6 | 0.93 |
|  | Haraz May 2012* | 66 ± 1.5 | 47 ± 3.3 | 2.8 | 10 | 0.96 |
|  | Haraz Dez 2012* | 74 ± 2.8 | 41 ± 4.9 | 2.7 | 10 | 0.90 |
|  | *Haraz all data* | *69 ± 2.5* | *37 ± 3.4* | *2.7* | *32* | *0.80* |
|  | Ammer May 2014 | 18 ± 8.0 | 160 ± 7.4 | 3.9 | 6 | 0.99 |
|  | Steinlach May 2014 | 29 ± 15 | 94 ± 40 | 3.5 | 9 | 0.44 |
|  | Steinlach July 2014a | 12 ± 12 | 127 ± 7.0 | 4.0 | 10 | 0.97 |
|  | Steinlach July 2014b | (≤ 0) | 89 ± 8.1 | -- | 8 | 0.95 |
|  | *Steinlach all data* | *17 ± 11* | *104 ± 7.0* | *3.8* | *27* | *0.89* |

*Nasrabadi T, Ruegner H, Sirdari ZZ, Schwientek M, Grathwohl P. Using total suspended solids (TSS) and turbidity as proxies for evaluation of metal transport in river water. Appl Geochemistry. 2016;68: 1-9. doi:10.1016/j.apgeochem.2016.03.003
